# Information-Theoretic Security and Privacy in Modern Data-Driven Systems

**DOI:** 10.3390/e28050483

**Published:** 2026-04-22

**Authors:** Mohamed Seif, Hua Sun

**Affiliations:** 1 Department of Electrical and Computer Engineering, Princeton University, Princeton, NJ 08544, USA; 2 Department Electrical Engineering, University of North Texas, Denton, TX 76203, USA; hua.sun@unt.edu

This Special Issue titled “Information-Theoretic Security and Privacy” brings together nine peer-reviewed contributions that span both fundamental theory and practical system design. The accepted papers cover a diverse range of topics, including private information retrieval, graph-based computation and analytics, differential privacy, federated and distributed learning, sequential inference, and system-level security mechanisms for emerging platforms such as the Internet of Things (IoT) and large language model (LLM) environments.

Overall, the contributions highlight how advances in this area arise from the interplay between information-theoretic modeling, algorithmic design, and application-driven constraints. Taken together, the papers confirm that information-theoretic approaches remain central to understanding and designing trustworthy, efficient, and scalable data-driven systems.

The published papers reflect several interconnected research directions:

- Information-theoretic privacy and coding, including variable-length coding under privacy constraints, cache-aided private updating, and graph-based secure and private linear computation, establishing new bounds and coding strategies for privacy–efficiency trade-offs.
- Privacy-preserving learning and inference, covering adaptive differential privacy for causal graph discovery, locally private sequential change detection, and differentially private optimization for imbalanced datasets, highlighting the role of adaptive and task-aware mechanisms.
- System-level security and practical privacy mechanisms, including authentication and key agreement protocols for IoT, robust Spectre attack detection under LLM workloads, and advanced secret image sharing schemes with improved access control and efficiency.

Across these directions, the contributions emphasize the need for structure-aware privacy design, where mechanisms are tailored to the underlying data, algorithms, and system constraints.

The papers in this Special Issue point toward several important future directions, including the development of unified frameworks that jointly address privacy, communication, and learning objectives, as well as scalable and adaptive mechanisms for high-dimensional, distributed, and adversarial environments. Bridging information-theoretic guarantees with practical deployment remains a central challenge, particularly in emerging applications involving AI-driven and resource-constrained systems.

We would like to thank all authors for their valuable contributions and the reviewers for their careful evaluations and constructive feedback. We are also grateful to the Editorial Office for their support throughout the review and publication process.

